# PD15 External Validation Of The Budget Impact Analysis Of The Drug Fingolimod In The Treatment Of Multiple Sclerosis

**DOI:** 10.1017/S0266462325101505

**Published:** 2025-12-29

**Authors:** Cesar Ricardo S. Campello, Márcia Gisele Costa, Marcia Pinto

## Abstract

**Introduction:**

This study aimed to externally validate the budget impact analysis of the drug fingolimod after its incorporation into the Brazilian Unified Health System, comparing real parameters arising from its incorporation in the treatment of relapsing-remitting multiple sclerosis with the estimates presented in the 2017 incorporation report.

**Methods:**

The study included the following:

- A survey of the target population to validate the demand measured by drug dispensing to patients with multiple sclerosis in the Brazilian Unified Health System;
- Validation of unit costs of drugs through purchases made by the Logistics Department of the Ministry of Health;
- Determination of market share within the defined time horizon based on drugs purchases;
- Estimation of direct administration costs; and
- Monitoring of drugs to compare estimated values with the real value.

**Results:**

Divergences were identified between the real-world data collected in relation to the study’s defined population and the market share of fingolimod over the studied time horizon. There was a significant increase in the fingolimod market share, with the largest market shares being in 2019 and 2020. The budget impact presented in the incorporation report was USD277,431,260.28, whereas the validated impact was USD194,955,442.76.

**Conclusions:**

The process of validating the 2017 budget impact analysis of fingolimod identified savings of around USD82.4 million relative to the value proposed for incorporation. The percentage of market share and drugs costs were the most divergent factors in relation to what had been proposed in the incorporation report.

